# Foliar Zinc-Selenium and Nitrogen Fertilization Affects Content of Zn, Fe, Se, P, and Cd in Wheat Grain

**DOI:** 10.3390/plants10081549

**Published:** 2021-07-28

**Authors:** Zdenko Lončarić, Vladimir Ivezić, Darko Kerovec, Andrijana Rebekić

**Affiliations:** 1 Department of Agroecology and Environmental Protection, Faculty of Agrobiotechnical Sciences Osijek, Josip Juraj Strossmayer University of Osijek, 31000 Osijek, Croatia; zloncaric@fazos.hr (Z.L.); vivezic@fazos.hr (V.I.); 2 Centre for Applied Life Sciences Healthy Food Chain Ltd., Vladimira Preloga 1, 31000 Osijek, Croatia; 3 Central Laboratory for Agroecology and Environmental Protection, Faculty of Agrobiotechnical Sciences Osijek, Josip Juraj Strossmayer University of Osijek, 31000 Osijek, Croatia; dkerovec@fazos.hr; 4 Department for Plant Production and Biotechnology, Faculty of Agrobiotechnical Sciences Osijek, Josip Juraj Strossmayer University of Osijek, 31000 Osijek, Croatia

**Keywords:** biofortification, in vitro bioaccessibility, iron, selenium, zinc

## Abstract

The grain yield and concentrations of Fe, Zn, Se, Cd, and P in two winter wheat genotypes and in vitro bioaccessibility of Fe and Zn under the effect of different nitrogen fertilization and Zn-Se foliar application were evaluated. The total grain Fe, Zn, and Se concentrations, as well as Fe and Zn concentrations, after in vitro digestion were under the strongest effect of foliar Zn-Se application. On the other hand, Fe and Zn bioaccessibility (%) were under the most substantial effect of genotype. Regarding the need to increase concentrations of essential micronutrients in wheat grain, foliar Zn-Se application is a reliable and accepted agricultural practice, but to improve mineral bioaccessibility in human nutrition, foliar Zn-Se application should be combined with the most responsive genotypes. For this reason, further research on the genotype specificity of wheat regarding micronutrient bioaccessibility should be carried out.

## 1. Introduction

Micronutrient deficiencies in the human population are a major public health challenge [1,2]. In general, they are much more common in underdeveloped countries, but some nutrient deficiencies, such as iron (Fe), zinc (Zn), and selenium (Se) are significantly prevalent in industrialized countries too [3,4]. Food is the primary source of nutrients for humans, and consumed food products should provide all of the necessary nutrients.

The main role of agriculture is to produce a sufficient quantity of food products but, besides quantity, a large emphasis is on the production of high quality and nutritionally rich products [5,6]. Unfortunately, it has been noted that some agricultural practices have had a significant role in the increase of micronutrient deficiencies over the past decades [7,8]. Some of the major reasons for the increment in micronutrient deficiencies are food production on poor soils, the “green revolution”, resulting in modern high yielding wheat cultivars (micronutrient dilution effect), unvaried rice, or wheat-based diets [9]. For example, wheat is a staple food for a large number of people, but wheat grain mineral density is lower than needed to satisfy the minimum daily requirements for humans [10]. On the other hand, at the same time, in order to solve this problem, different agricultural practices such as biofortification have been developed [9]. Biofortification (agronomic and genetic) is accepted as a successful approach for increasing grain nutrients to the extent that it is limited by genotypic specificity [11,12,13]. Besides, micronutrients from wheat grain, especially iron and zinc, are poorly available in humans due to their bond to phytate [14]. Consequently, agricultural practices developed to increase grain mineral density should also have a positive effect on their bioaccessibility. Bioaccessibility represents the amount of micronutrient that can be released from its matrix into the gastrointestinal tract and become available for absorption. Thus, the goal of biofortification and other agricultural practices should not be only to increase micronutrient concentrations in grain, but rather to increase their bioaccessibility from grain. Furthermore, recently, there has been much interest in ancient wheat, such as einkorn, emmer, and spelt wheat, as a source of biodiversity in terms of chemical composition and better nutritional properties in comparison to modern wheat [15,16]. Additionally, it has been shown that the ancestors of cultivated wheat that belong to genus *Aegilops*, such as Ae.*ventricosa* Tausch, are naturally rich in Zn and Fe [17,18], so it would be beneficial to evaluate them and include them in research in order to select varieties with a higher ability to accumulate these microelements in grain [19]. This study aimed to investigate the effect of location, genotype, N fertilization, and Zn-Se foliar application on grain yield and Fe, Zn, Se, P, and Cd concentration as well as on in vitro bioaccessibility of Fe and Zn from wheat grain.

## 2. Materials and Methods

### 2.1. Location and Weather Conditions

A field experiment has been carried out at the agricultural fields of Novi Agrar at location Josipin Dvor (JD) (45.51° N, 18.67° E) and location Ernestinovo (E) (45.44° N, 18.67° E), situated in Eastern Croatia. Given that the experimental locations are only 9 km apart, the elevation is similar at both locations and is in the range between 80–90 m above sea level. According to the Köppen climate classification, the climate at the experimental locations is a temperate humid climate with warm summer (Cfb). During the vegetation season (October–July), the total annual rainfall was 586 mm, which is 21% above the long-term (1961–1990) average (LTA). Although there was enough rainfall (in total) during the vegetation season, the precipitation was unevenly distributed over the months. For example, there was no precipitation in January (0 mm) in contrary to LTA where the amount of rainfall in January was 59 mm. On the other hand, there was 159 mm of precipitation in May, which is almost twice as much as the LTA (74 mm). However, during the vegetation, between sowing and tillering stage, distribution of precipitation over the months was even, with only 24% less rainfall in comparison to the LTA in continental Croatia. From the tillering stage to full maturity there was 45% more precipitation in comparison to the LTA. 

In the vegetation season (October–July), the average air temperature was 10.1 °C, which is 2.3 °C above the LTA for the same period. Additionally, average monthly temperatures for each month during the vegetation were higher than the LTA temperatures. Winter was mild; the coldest month was December, with an average temperature of 1.6 °C (0.6 °C above the LTA for December). Moreover, January was 4.5 °C and February 3.4 °C warmer than the LTA temperatures in January and February.

### 2.2. Soil Analysis

The soil samples were prepared for chemical analyses according to [20] ISO 10390 procedure for pretreatment of samples for physicochemical analyses. Soil pH was measured in a 1:5 (volume fraction) suspension of soil in water (pH H_2_O) and 1 mol L^−1^ potassium chloride solution (pH KCl) according to ISO 10390 procedure [18]. Phosphorus and potassium were extracted by AL-acetic acid method [21] and expressed as P_2_O_5_ and K_2_O in mg 100 g^−1^ of soil. The humus content (soil organic matter) was determined using the determination of organic carbon by sulfochromic oxidation, as recommended in the ISO 14235 [22] procedure (ISO 1998). Soil properties at experimental locations are shown in Table 1.

The Fe, Zn, and Cd in the soil samples were extracted by aqua regia [23], and this fraction was considered as total soil content and expressed in mg kg^−1^. The soil samples were digested at 210 °C for 60 min in a microwave oven (CEM Mars 6). Fe, Zn, and Cd were extracted by ethylenediaminetetraacetic acid (EDTA) as plant available fraction in soil (Table 2).

### 2.3. Wheat Genotypes and Treatments

Winter wheat (*Triticum aestivum* L.) genotypes Srpanjka and Renata were sown in October and harvested in July at both locations. All agrotechnical measures were carried out by standard procedures in winter wheat production in this area. Nitrogen (N) treatment was applied as N fertilization in the concentration of 0 (control treatment), 105 (reduced fertilization), 140 (optimal fertilization), and 180 kg N ha^−1^ (excessive fertilization). The N was applied in the form of urea before sowing. Foliar application of zinc and selenium (Zn-Se) were carried out at the beginning of flowering (Feekes 10.51). A combination of 1.5 kg Zn ha^−1^ in the form of ZnSO_4_ and 10 g Se ha^−1^ in the form of Na_2_SeO_4_ were applied as a foliar treatment (Zn-Se 1), as opposed to the control treatment, which did not receive a foliar application (Zn-Se 0). All applied foliar solutions contained surfactant (Tween 20).

### 2.4. Sample Preparation and Laboratory Analysis

Both genotypes at both locations were harvested at full maturity. Grain samples were dried at 70 °C for 72 h and milled in a micronutrient free mill (Retsch RM200, Haan, Germany) into whole wheat flour containing all milling fractions. A 1 g sample of milled grain was digested with 9 mL 65% (*v*/*v*) HNO_3_ and 2 mL 30% (*v*/*v*) H_2_O_2_ in microwave vessels (CEM Mars 6, Matthews, NC, US), according to [24]. Samples were digested for 60 min at 180 °C. After digestion, the cooled sample solution was filtered through a double filter paper and transferred into a 50 mL graduated flask, which was filled with deionized water up to the volume of 50 mL. For the Se determination, 5 mL of concentrated HCl was added to the cooled digest to reduce Se^6+^ to Se^4+^. Concentrations of Fe, Zn, Se, Cd, and P were determined by inductively coupled plasma-optical emission spectrometry (ICP-OES) technique (Perkin Elmer–Optima 2100 DV, Überlingen, Germany) using an internal pooled plasma control. Reference material (Rice flour, IRMM-804, Sample No. 0533, European Commission, Joint Research Centre, Institute for Reference Materials and Measurements, Geel, Belgium) was prepared in the same way as the grain samples. 

In vitro digestion was carried out according to [25]. Concentrations of Zn and Fe in the supernatant after in vitro digestion were determined by ICP-OES technique. The percentage of bioaccessibility (B (%)) in the samples was calculated as follows:

B (%) = (concentration after in vitro digestion × 100)/(concentration in whole grain)

### 2.5. Statistical Analysis

The experiment was designed as a completely randomized block design with four factors (location, genotype, N fertilization, and Zn-Se foliar application) in four replicates. Factorial four-way (for grain yield and Fe, Zn, Se, P, and Cd concentration in grain) and three-way analysis of variance (for in vitro bioaccessibility and % of bioaccessibility of Fe and Zn) were carried out. The partial eta square coefficient was used as a measure of effect size. Mean values were compared using Fishers least significant difference test (LSD test). Only differences significant at *p* < 0.01 were considered. The relationships between the examined traits were obtained by Pearson’s correlation coefficient (r) at a significance level of *p* < 0.01. All statistical analyses were done by Enterprise Guide 5.1. of the SAS System for Windows (Copyright© 2021 by SAS Institute Inc., Cary, NC, USD, All Rights Reserved).

## 3. Results and Discussion

### 3.1. The Effect of Location on Grain Yield and Fe, Zn, Se, P, and Cd Concentration

In plant production, soil, topographic and vegetation characteristics, and weather conditions are essential environmental factors that influences a plant during all stages of development [26,27] and have a substantial effect on the yield and mineral composition of grain [28,29]. Among other things, the concentration of an element in soil and its phytoavailability, soil pH, the content of organic matter, clay and calcium carbonate, microbiological activity in the rhizosphere, soil moisture, and temperature, as well as climate, are factors that determinate accumulation of elements in different plant parts. 

In the present study, grain yield (Table 3) and concentrations of Fe, Zn, Se, and Cd in wheat grain were under the significant effect of location (Table 4).

The Fe, Zn, and Se concentrations in grain were higher at location E, at 6.9%, 7.3%, and 27%, respectively (Table 4), while grain yield was 8% lower at location E in comparison to yield at location JD (Table 3). On the contrary, grain Cd concentration was 2.92-fold higher at location JD compared to E (Table 4). Higher soil Cd concentration can increase grain Cd concentration in comparison to uncontaminated soil [30]. At location JD, total soil Cd concentration was only 3%, and EDTA extractable Cd was 15% higher compared to location E (Table 2). That does not seem substantial enough to increase grain Cd for 2.92-fold alone. This finding indicates the importance of other factors (soil pH, soil P, and humus content) or their interactions on grain Cd concentration that is a highly complex trait. Besides, soil total and EDTA extractable concentrations of other elements were also higher at location JD (Table 2), but grain concentration of elements was lower compared to location E (Table 4). At the same time, humus content (organic matter) that can enhance nutrient availability to plants indirectly, through the increase in CEC complex [28], was twice as high at location E (Table 2). The higher soil Cd content at location JD could affect the phytoavailability of other elements from the soil as well as the abundance of soil microorganisms [31] and create unfavorable conditions for the adoption of other elements.

### 3.2. The Effect of Genotype on Grain Yield and Fe, Zn, Se, P and Cd Concentration

The Fe, Zn, Se, P, and Cd concentrations in grain were under the significant effect of wheat genotype (Table 4). The genotype Renata accumulated higher amounts of all examined elements in grain in comparison to genotype Srpanjka (Table 4). The most considerable differences between genotypes were determined in grain Se (26%) and Cd (20%) concentrations. The P and Fe concentrations were 10%, and Zn concentration were 13% higher in Renata than in Srpanjka. Two genotypes are not enough to discuss genotypic specificity, but the differences between them are in accordance with previously published results on the genotype specificity of winter wheat regarding the accumulation of Fe, Zn, Se, and Cd in the grain [32,33,34]. Both genotypes included in this research are Croatian genotypes, released in the year 1989 (Srpanjka) and 2006 (Renata). There are some similarities between genotypes in agronomic traits due to the genotype Renata pedigree (Srpanjka was one of Renata’s parents) [35]. Although these genotypes differ in quality traits [36], they are similar in grain yield (as confirmed by this study) (Table 3) and plant height, two traits that are very often related to the mineral content of grain. Grain mineral concentration is in significant negative relation to grain yield and harvest index, indicating that high-yielding cultivars have a decreased ability to accumulate minerals in grain. Lower accumulation of minerals in the grain of modern cultivars could be a result of a decrease in a plant height, which is in relation to the length of the root system [37]. However, it seems that genotype specificity in the accumulation of minerals in grain is, besides the effect of the shorter root system, also under the impact of other factors [38]. In some older varieties, grain Zn and grain size were in positive correlation, indicating that it could be possible to combine high grain yield and high grain Zn. On the global scale, grain Zn concentration varies between 20.4 and 30.5 mg kg^−1^ [39]. Average Zn and Fe concentrations in the grain of both genotypes (Table 4) are in accordance with grain Fe and Zn concentrations in cultivated wheat obtained by other authors [10,40].

Se concentration in the grain varies between 5 and 720 μg kg^−1^, and that variation is mostly influenced by spatial variation in soil Se. Average Se concentration in the grain obtained in this study was consistent with [41] but lower than the targeted level of 400 μg kg^−1^. Similarly, reported average concentrations of Fe and Zn in others as well in this study were far below the targeted concentration of 40 mg kg^−1^ [42]. Accordingly, at this moment, the vast majority of cultivars used for grain production do not meet the targeted level for grain Zn, Fe, or Se concentration, indicating that foliar application of Zn and Se should be an important strategy for the increment of these elements in grain.

### 3.3. The effect of Foliar Application of Zn and Se on Grain Yield Fe, Zn, Se, P, and Cd Concentrations

Biofortification of wheat grain through foliar application of Zn and Se alone or in combination with other elements is an accepted method for the increase of these elements in the grain. The effectiveness of foliar application depends on a large number of factors, but mostly on the concentrations and combination of applied elements and the time of application. The effect of foliar application of Zn and Zn combined with P or N on Zn concentration in grain has been investigated by [43], who proposed a combination of foliar Zn and N application as a potential strategy for the improvement of Zn bioavailability. In this study, foliar application of Zn-Se increased Zn concentration in grain by 46% (Table 4) in comparison to the control treatment, but even with such a significant increase in comparison to non-foliar application, grain Zn concentration still did not reach a targeted level of 40 mg Zn kg^−1^ (Table 5). An increase of 10.01 mg kg^−1^ in average grain Zn concentration on Zn-Se foliar application is consistent with [39], who reported an increase in average grain Zn concentration for 10.5 mg kg^−1^ by the foliar application of Zn. A foliar application of Zn is usually carried out at the begging of the flowering stage. Given that Zn translocation from leaves to grain contributes more to the grain Zn than Zn uptake during the grain filling [44], leaves and flag leaf are vital pools of Zn for accumulation in grain. Besides, wheat grain could be a valuable source of Se if Se concentration in grain would be around 400 ng g^−1^. Daily intake of Se has declined in the past decades due to consumption of food poor in Se. Low soil Se and low EDTA extractable Se are one of the reasons why Se concentrations in grains are so small. Foliar application of Zn-Se increased grain Se concentration 6.6-fold in comparison to no foliar treatment (Table 4). In addition, grain Se and Zn were in strong positive correlation (r = 0.85; *p* < 0.01). A similar increase (6-fold) in grain Se was observed in [11], but only in treatments where Se was combined with Zn, indicating that the interaction of Zn and Se should be further studied to reveal the mechanisms responsible for their accumulation in grain. 

Furthermore, Se is essential to humans, but not to vascular plants, so a concentration of Se applied with an intent to increase Se concentration in wheat grain should not induce phytotoxicity symptoms in plants. Broadley [45] reported that an amount of 100 g Se ha^−1^ did not cause any visible symptoms or decline in yield in comparison to the control treatment. Applied Se (in combination with Zn) increased average Se concentration by 0.196 mg Se kg^−1^ grain (which is still lower than the targeted value). Given that 10 times higher Se concentration [45] did not cause phytotoxicity symptoms in wheat plants, it would be recommended to carry out a study with increasing concentrations of Se with the aim to achieve a desirable Se concentration in the grain. 

Besides, foliar Zn-Se application had a significant, but negative, effect on Fe (−11%), P (−4%), and Cd (−28%) concentration in grain (Table 4). There are many reports on the interactions between elements [29,46,47], but there is no unified conclusion on their interaction, mostly because of important genotype effects, heterogeneous phytoavailability of elements from the soil, and differential environmental conditions and agricultural practices. The addition of the Zn to the soil increases its accumulation in the grain and decreases the accumulation of Cd in grain [48], most probably because of its competition with Zn for usage of the same adoption mechanisms. 

### 3.4. The Effect of Nitrogen Fertilization on Grain Yield Fe, Zn, Se, P, and Cd Concentrations

A nitrogen (N) is a key macronutrient essential for the production of a photosynthetically active canopy and storage proteins in the grain [49]. To provide enough N for plants, N fertilization is a conventional agricultural practices. In this study, the effect of four different levels of N on grain Fe, Zn, Se, P, and Cd concentration was investigated. Grain yield (Table 3) and all examined elements were under the significant effect of N fertilization (Table 4). The grain yield (Table 3) and Fe, Zn, Se, and Cd concentrations increased under the effect of N fertilization, while P concentration decreased (Table 4). The Zn, Se, and Cd concentration in grain were lowest at the control treatment and significantly lower than on all other N treatments (Table 4). Similarly to [50], N concentration of 105 kg ha^−1^ was equally effective as higher concentrations because there were no significant differences between N105 and other higher N concentrations. Furthermore, in contrast to our findings, where N and foliar Zn-Se in combination did not have a significant effect on any of the examined elements (Table 3), [43] reported that foliar Zn combined with N increased Zn concentration and bioavailability in grain.

Based on partial eta coefficients, in this study, Se (0.93), Zn (0.83), and Fe (0.29) grain concentrations were under the strongest effect of foliar Zn-Se application in comparison to the other factors included in this study. However, grain P was under the most substantial effect of genotype (0.49), while Cd (0.75) was under the strongest effect of location. Similarly, [51] found that Fe and Zn concentrations in winter wheat grain were mostly under the effect of genotype and P concentration was mainly under the effect of location, while Cd concentration in grain was under the effect of both factors, suggesting that accumulation of elements is under the influence of various factors that are element-specific.

### 3.5. In Vitro Bioaccessibility of Zn and Fe

Concentrations of Zn (partial eta coefficient 0.47) and Fe (partial eta coefficient 0.27), after in vitro digestion, were under the strongest effect of foliar Zn-Se application, followed by the effect of genotype. At the same time, genotype effect was the most important factor for Fe (0.26) and Zn (0.40) bioaccessibility (%), followed by a foliar Zn-Se application for Zn (0.18) and nitrogen fertilization for Fe (0.23) bioaccessibility (%) (Table 5). Lower bioaccessibility (%) of Fe and Zn in the genotype Renata in comparison to Srpanjka could be related to higher grain P content compared to Srpanjka (Table 5). Total P content in grain is highly correlated to grain phytic acid (r = 0.96). Phytic acid is the storage form of P in plant seed, and it is estimated that 60–80% of seed P is bound in phytic acid [52,53]. 

Besides, phytic acid is an antinutrient that decreases the bioavailability of dietary nutrients in humans and monogastric animals [54]. As an illustration, the cultivar Renata had a higher grain P concentration that is highly correlated to the content of phytic acid. Based on that, we can assume that Renata had lower nutrient bioaccessibility in comparison to Srpanjka due to the higher content of phytic acid. However, for a more precise estimation, a trivariate model of Zn absorption [55] or laboratory measurement of phytic acid should be carried out.

The N fertilization did not have a significant effect on Fe and Zn concentration after in vitro digestion, but Fe bioaccesibility (%) was under the significant negative effect of N fertilization. A decrease in Fe bioaccessibility was 10% between the control treatment (0 kg N ha^−1^) and excessive fertilization (180 kg N ha^−1^). Although there was no significant effect of N fertilization on Zn bioaccessibility (%), a decreasing pattern was recorded (7% difference between 0 N and 180 N) in spite of an increase in grain Zn concentration under the increased N fertilization. On the contrary, based on the [phytate]:[Zn] ratio in flour and whole grain and the trivariate model of Zn absorption, [43] found that a combination of N and foliar Zn application increases Zn concentration and Zn bioaccessibility.

## 4. Conclusions

Wheat production is under the substantial effect of environmental factors that cannot be entirely controlled. Factors that are controllable should be used to produce high-quality products. N fertilization is commonly used in wheat production to increase grain yield. In a term of increase in the mineral concentration of grain, N fertilization has a significant positive effect on grain Fe, Zn, and Cd concentrations and a significant adverse effect on Fe bioaccessibility (%). Foliar Zn-Se application has a substantial positive effect on Zn and Se grain concentration, while grain Fe, P, and Cd concentrations decreased under foliar Zn-Se application. Furthermore, Zn-Se foliar application increased Zn in vitro bioaccessibility (mg kg^−1^). Generally speaking, grain Fe, Zn, and Se concentrations were under the strongest effect of foliar Zn-Se application, Fe bioaccessibility was under the strongest effect of N fertilization, while Zn bioaccessibility was mostly affected by genotype. Because the bioaccesibility of Zn is under substantial effect of genotype, future work should be focused on the research of Zn rich genotypes or ancestor wheat as a material for research of mineral bioaccesibility. From the perspective of human nutrition, the bioaccessibility of minerals in wheat grain is an important trait. Ancient wheat and modern genotypes differ in their ability to accumulate minerals as well as in mineral bioaccessibility in grain, so future work should be focused on the selection of the most suitable genotypes for the research on bioaccessibility.

## Figures and Tables

**Table 1:** Soil properties at location Josipin Dvor (JD) and Ernestinovo (E).

Location	pH (H_2_O)	pH (KCl)	AL-P_2_O_5_ (mg 100 g^−1^)	AL-K_2_O (mg 100 g^−1^)	Humus (%)	CaCO_3_ (%)
JD	7.60	6.88	25.58	24.72	2.14	1.44
E	8.44	7.49	15.41	18.28	4.27	7.82

**Table 2:** Total soil concentrations and EDTA extractable concentrations of Fe, Zn, and Cd at experimental locations.

	Total Concentration of Elements in Soil (mg kg^−1^)			EDTA Extractable Elements in Soil (mg kg^−1^)		
Location	Fe	Zn	Cd	Fe	Zn	Cd
JD	28155	89.45	0.34	56.88	12.38	0.15
E	26850	54.00	0.33	50.98	1.54	0.13

JD, Josipin Dvor; E, Ernestinovo.

**Table 3:** Average values of grain yield (t ha^−1^) at different locations, genotypes, foliar Zn-Se, and nitrogen application (kg ha^−1^).

Location		Genotype		Foliar Zn-Se		Nitrogen	
**JD**	6.38 ± 1.91 ^b^	**R**	6.08 ± 1.71a	**0**	6.18 ± 1.69 ^a^	**0**	3.92 ± 0.47 ^a^
						**105**	6.42 ± 1.11 ^b^
**E**	5.91 ± 1.33 ^a^	**S**	6.21 ± 1.61a	**1**	6.11 ± 1.63 ^a^	**140**	7.12 ± 1.11 ^c^
						**180**	7.11 ± 1.19 ^c^
F ^a^ = 6.61 *		F = 0.49		F = 0.16		F = 71.23 **	

Each value is represented as mean ± SD (n = 4). Values in a column followed by a different letter are significantly different (difference between levels of the same factor). F values for each source of variation are shown and marked as ** (*p* < 0.01) or * (*p* < 0.05). JD, Josipin Dvor; E, Ernestinovo; R, Renata; S, Srpanjka, 0, Zn-Se 0; 1, Zn-Se 1. ^a^ According to factorial ANOVA, two, three, and four factorial interactions did not have a significant effect on the examined trait, so their F values are not shown in the table.

**Table 4:** Average values of Fe, Zn, Se, P, and Cd concentration in grain (mg kg^−1^) at different locations, genotypes, foliar Zn-Se applications, and levels of nitrogen fertilization.

Treatments	Fe	Zn	Se	P	Cd
**Location**					
Josipin Dvor	27.1 ± 3.99 ^b^	25.6 ± 6.17 ^b^	0.112 ± 0.09 ^b^	3498 ± 300 ^a^	0.041 ± 0.014 ^b^
Ernestinovo	29.1 ± 5.13 ^a^	27.6 ± 6.18 ^a^	0.153 ± 0.11 ^a^	3528 ± 304 ^a^	0.014 ± 0.011 ^a^
**Genotype**					
Renata	29.5 ± 5.54 ^b^	28.3 ± 5.75 ^b^	0.148 ± 0.11 ^b^	3685 ± 297 ^b^	0.030 ± 0.012 ^b^
Srpanjka	26.7 ± 3.10 ^a^	24.9 ± 6.28 ^a^	0.117 ± 0.09 ^a^	3341 ± 187 ^a^	0.025 ± 0.010 ^a^
**Foliar Zn-Se**					
Zn-Se 0	29.8 ± 4.85 ^a^	21.6 ± 3.66 ^b^	0.035 ± 0.02 ^a^	3587 ± 316 ^b^	0.032 ± 0.016 ^b^
Zn-Se 1	26.4 ± 3.85 ^b^	31.6 ± 3.75 ^a^	0.231 ± 0.06 ^b^	3439 ± 270 ^a^	0.023 ± 0.017 ^a^
**Nitrogen**					
0	26.4 ± 3.60 ^a^	24.3 ± 5.70 ^a^	0.122 ± 0.10 ^a^	3594 ± 329 ^a^	0.022 ± 0.015 ^a^
105	27.1 ± 3.71 ^a^	26.7 ± 6.56 ^b^	0.138 ± 0.10 ^b^	3480 ± 270 ^b^c	0.028 ± 0.017 ^b^
140	29.0 ± 5.28 ^b^	27.6 ± 6.39 ^b^	0.143 ± 0.12 ^b^	3540 ± 304 ^ab^	0.029 ± 0.017 ^b^
180	29.9 ± 5.24 ^b^	27.8 ± 5.93 ^b^	0.129 ± 0.10 ^a b^	3437 ± 291c	0.030 ± 0.019 ^b^
**Source of Variation ^a^**					
Location (L)	13.27 **	19.02 **	53.08 **	0.7	290 **
Genotype (G)	26.13 **	54.7 **	30.66 **	91.86 **	9.18 **
Foliar Zn-Se (F)	39.42 **	475 **	1206 **	17.02 **	28.06 **
Nitrogen (N)	8.6 **	12.07 **	2.83	3.66	6.34 **
L × G	21 **	5.72	1.56	0.00	0.61
L × F	0.03	0.08	4.37	0.00	13.70 **
L × N	1.87	0.89	0.21	4.85 **	0.75
G × F	30.04 **	2.30	13.66	14.04 **	12.91 **
G × N	1.22	0.56	1.73	2.81	1.39
F × N	2.05	1.24	1.8	1.44	1.14

Each value is represented as mean ± SD (n = 4). Values in a column followed by a different letter are significantly different according to LSD test (difference between levels of the same factor); F values for each source of variation are shown and marked as ** *p* < 0.01 ^a^ According to factorial ANOVA, three and four factorial interaction did not have a significant effect on the examined traits, so their F values are not shown in the table.

**Table 5:** In vitro bioaccessible concentrations of Fe and Zn (mg kg^−1^) and percentage of Fe and Zn bioaccessibility (%) from wheat grain.

Treatments	In Vitro Fe	In Vitro Zn	Fe (%)	Zn (%)
**Genotype**				
Renata	8.87 ± 1.24 ^b^	4.41 ± 0.92 ^b^	28.5 ± 4.30 ^a^	15.5 ± 2.62 ^b^
Srpanjka	8.38 ± 0.68 ^a^	4.93 ± 0.93 ^a^	31.9 ± 3.50 ^a^	19.0 ± 2.56 ^a^
**Foliar Zn-Se**				
Zn-Se 0	9.03 ± 1.10 ^b^	4.09 ± 0.80 ^b^	29.8 ± 3.83 ^a^	18.3 ± 3.40 ^b^
Zn-Se 1	8.22 ± 0.56 ^a^	5.26 ± 0.72 ^a^	30.6 ± 4.66 ^a^	16.2 ± 2.51 ^a^
**Nitrogen**				
0	8.54 ± 0.68 ^a^	4.57 ± 0.96 ^a^	32.6 ± 4.07 ^a^	18.2 ± 2.55 ^a^
105	8.51 ± 0.55 ^a^	4.67 ± 0.93 ^a^	30.7 ± 3.50 ^ab^	17.1 ± 3.55 ^a^
140	8.81 ± 1.13 ^a^	4.76 ± 1.14 ^a^	28.2 ± 3.70c	16.8 ± 3.30 ^a^
180	8.65 ± 1.31 ^a^	4.69 ± 0.83 ^a^	29.3 ± 4.69 ^b^	16.9 ± 3.66 ^a^
**Sources of Variation ^a^**	**F Value**			
Genotype (G)	6.56 **	8.53 **	16.63 **	32.42 **
Foliar Zn-Se (F)	17.7 **	42.3 **	1.00	10.46 **
Nitrogen (N)	0.49	0.19	5.03 **	0.97

Each value is represented as mean ± SD (n = 3). Values in a column followed by different letters are significantly different among different levels of the same factor (*p* < 0.01). F values for each source of variation are shown and marked as ** (*p* < 0.01). ^a^ According to factorial ANOVA, two and three factorial interaction did not have a significant effect on examined traits, so their F values are not shown in the table.

## References

[1] Bhutta Z.A., Salam R.A., Das J.K. (2013). Meeting the challenges of micronutrient malnutrition in the developing world. Br. Med. Bull..

[2] Tulchinsky T.H. (2010). Micronutrient deficiency conditions: Global health issues. Public Health Rev..

[3] Whatham A., Bartlett H., Eperjesi F., Blumenthal C., Allen J., Suttle C., Gaskin K. (2008). Vitamin and mineral deficiencies in the developed world and their effect on the eye and vision. Ophthalmic Physiol. Opt..

[4] Stoffaneller R., Morse N.L. (2015). A review of dietary selenium intake and selenium status in Europe and the Middle East. Nutrients.

[5] Demment M.W., Young M.M., Sensenig R.L. (2003). Providing micronutrients through food-based solutions: A key to human and national development. Nutr. J..

[6] Gillespie S., van den Bold M. (2017). Agriculture, food systems, and nutrition: Meeting the challenge. Glob. Chall..

[7] Tan Z.X., Lal R., Wiebe K.D. (2005). Global soil nutrient depletion and yield reduction. J. Sustain. Agric..

[8] Lal R. (2009). Soil degradation as a reason for inadequate human nutrition. Food Secur..

[9] Welch R.M., Graham R.D., Cakmak I. (2013). Linking Agricultural Production Practices to Improving Human Nutrition and Health.

[10] Gao X., Mohr R.M., McLaren D.L., Grant C.A. (2011). Grain cadmium and zinc concentrations in wheat as affected by genotypic variation and potassium chloride fertilization. F. Crop. Res..

[11] Velu G., Ortiz-Monasterio I., Cakmak I., Hao Y., Singh R.P. (2014). Biofortification strategies to increase grain zinc and iron concentrations in wheat. J. Cereal Sci..

[12] Bouis H.E., Welch R.M. (2010). Biofortification—A sustainable agricultural strategy for reducing micronutrient malnutrition in the global south. Crop Sci..

[13] Germ M., Pongrac P., Regvar M., Vogel-Mikuš K., Stibilj V., Jaćimović R., Kreft I. (2013). Impact of double Zn and Se biofortification of wheat plants on the element concentrations in the grain. Plant Soil Environ..

[14] Borrill P., Connorton J.M., Balk J., Miller A.J., Sanders D., Uauy C. (2014). Biofortification of wheat grain with iron and zinc: Integrating novel genomic resources and knowledge from model crops. Front. Plant Sci..

[15] Van Slageren M.W. (1994). Wild Wheats: A Monograph of Aegilops and Amblyopyrum (Jaub. et Spach) Eig (Poaceae).

[16] Perrino E.V., Wagensommer R.P., Medagli P. (2014). The genus *Aegilops* (Poaceae) in Italy: Taxonomy, geographical distribution, ecology, vulnerability and conservation. Syst. Biodivers.

[17] Dinu M., Whittaker A., Pagliai G., Benedettelli S., Sofi F. (2018). Ancient wheat species and human health: Biochemical and clinical implications. J. Nutr. Biochem..

[18] Perrino E.V., Wagensommer R.P. (2021). Crop Wild Relatives (CWR) Priority in Italy: Distribution, Ecology, In Situ and Ex Situ Conservation and Expected Actions. Sustainability.

[19] Abenavoli L., Milanovic M., Procopio A.C., Spampinato G., Maruca G., Perrino E.V., Mannino G.C., Fagoonee S., Luzza F., Musarella C.M. (2021). Ancient wheats: Beneficial effects on insulin resistance. Minerva Med..

[20] International Standard Organization (2005). Soil Quality: Determination of pH (ISO 10390:2005), (n.d.).

[21] Egner W.R., Riehm H., Domingo H. (1960). Investigations on the chemical soil analysis as a basis for assessing the soil nutrient status. II: Chemical extraction methods for phosphorus and potassium determination. K. Lantbr. Ann..

[22] International Standard Organization (1998). Soil Quality: Determination of Organic Carbon by Sulfochromic Oxidation. (ISO 14235:1998), (n.d.).

[23] International Standard Organization (1995). Soil Quality: Extraction of Trace Elements Soluble in Aqua Regia. (ISO 11466:1995), (n.d.).

[24] Kingstone L.B., Lassie H.M.S. (1986). Microwave Energy for Acid Decomposition at Elevated Temperatures and Pressures Using Biological and Botanical Samples. Anal. Chem..

[25] Kiers J.L., Nout R.M.J., Rombouts F.M. (2000). In vitro digestibility of processed and fermented soya bean, cowpea and maize. J. Sci. Food Agric..

[26] Perrino E.V., Valerio F., Gannouchi A., Trani A., Mezzapesa G. (2021). Ecological and Plant Community Implication on Essential Oils Composition in Useful Wild Officinal Species: A Pilot Case Study in Apulia (Italy). Plants.

[27] Valerio F., Mezzapesa G.N., Ghannouchi A., Mondelli D., Logrieco A.F., Perrino E.V. (2021). Characterization and antimicrobial properties of essential oils from four wild taxa of Lamiaceae family growing in Apulia. Agronomy.

[28] Zeng F., Ali S., Zhang H., Ouyang Y., Qiu B., Wu F., Zhang G. (2011). The influence of pH and organic matter content in paddy soil on heavy metal availability and their uptake by rice plants. Environ. Pollut..

[29] Liu J.G., Liang J.S., Li K.Q., Zhang Z.J., Yu B.Y., Lu X.L., Yang J.C., Zhu Q.S. (2003). Correlations between cadmium and mineral nutrients in absorption and accumulation in various genotypes of rice under cadmium stress. Chemosphere.

[30] Rebekić A., Lončarić Z. (2016). Genotypic difference in cadmium effect on agronomic traits and grain zinc and iron concentration in winter wheat. Emir. J. Food Agric..

[31] Tudoreanu L., Phillips C.J.C. (2004). Empirical models of cadmium accumulation in maize, rye grass and soya bean plants. J. Sci. Food Agric..

[32] Zhao F.J., Su Y.H., Dunham S.J., Rakszegi M., Bedo Z., McGrath S.P., Shewry P.R. (2009). Variation in mineral micronutrient concentrations in grain of wheat lines of diverse origin. J. Cereal Sci..

[33] Souza G.A., Hart J.J., Carvalho J.G., Rutzke M.A., Albrecht J.C., Guilherme L.R.G., Kochian L.V., Li L. (2014). Genotypic variation of zinc and selenium concentration in grains of Brazilian wheat lines. Plant Sci..

[34] Guttieri M.B., Baenziger M.J., Frels S.P., Carver K., Arnall B., Waters B. (2015). Variation for Grain Mineral Concentration in a Diversity Panel of Current and Historical Great Plains Hard Winter Wheat Germplasm. Crop Sci..

[35] Petrović S., Marić S., Čupić T., Rebekić A., Rukavina I. (2017). Assessment of molecular and phenotypic diversity among winter wheat cultivars. Genetika.

[36] Španić V., Drezner G., Dvojković K., Horvat D. (2016). Traits of 25 Winter Wheat Varieties Grown in Croatia in the Last 100 Years. Agron. J..

[37] Fan M.S., Zhao F.J., Fairweather-Tait S.J., Poulton P.R., Dunham S.J., McGrath S.P. (2008). Evidence of decreasing mineral density in wheat grain over the last 160 years. J. Trace Elem. Med. Biol..

[38] Yilmaz O., Kazar G.A., Cakmak I., Ozturk L. (2017). Differences in grain zinc are not correlated with root uptake and grain translocation of zinc in wild emmer and durum wheat genotypes. Plant Soil.

[39] Chen X.P., Zhang Y.Q., Tong Y.P., Xue Y.F., Liu D.Y., Zhang W., Deng Y., Meng Q.F., Yue S.C., Yan P. (2017). Harvesting more grain zinc of wheat for human health. Sci. Rep..

[40] Cakmak I., Ozkan H., Braun H.J., Welch R.M., Romheld V. (2000). Zinc and iron concentrations in seeds of wild, primitive, and modern wheats. Food Nutr. Bull..

[41] Mao H., Wang J., Wang Z., Zan Y., Lyons G., Zou C. (2014). Using agronomic biofortification to boost zinc, selenium, and iodine concentrations of food crops grown on the loess plateau in China. J. Soil Sci. Plant Nutr..

[42] Bouis H.E., Hotz C., McClafferty B., Meenakshi J.V., Pfeiffer H. (2011). Biofortification: A New Tool to Reduce Micronutrient Malnutrition. Food. Nutr. Bull..

[43] Li M., Wang S., Tian X., Zhao J., Li H., Guo C., Chen Y., Zhao A. (2015). Zn distribution and bioavailability in whole grain and grain fractions of winter wheat as affected by applications of soil N and foliar Zn combined with N or P. J. Cereal Sci..

[44] Stomph J.T., Jiang W., Struik P.C. (2009). Zinc biofortification of cereals: Rice differs from wheat and barley. Trends Plant Sci..

[45] Broadley M.R., Alcock J., Alford J., Cartwright P., Foot I., Fairweather-Tait S.J., Hart D.J., Hurst R., Knott P., McGrath S.P. (2010). Selenium biofortification of high-yielding winter wheat (*Triticum aestivum* L.) by liquid or granular Se fertilisation. Plant Soil.

[46] Chen F., Dong J., Wang F., Wu F., Zhang G., Li G., Chen Z., Chen J., Wei K. (2017). Identification of barley genotypes with low grain Cd accumulation and its interaction with four microelements. Chemosphere.

[47] Chakroun H.K., Souissi F., Bouchardon J., Souissi R., Faure O., Remon E., Abdeljaoued S. (2010). Transfer and accumulation of lead, zinc, cadmium and copper in plants growing in abandoned mining-district area. Afr. J. Environ. Sci. Technol..

[48] Nan Z., Zhao C., Li J., Chen F., Sun W. (2002). Relations Between Soil Properties and Selected Heavy Metal Concentrations in Spring Wheat (*Triticum aestivum* L.) Grown in Contaminated Soils. Water Air Soil Pollut..

[49] Hawkesford M.J. (2014). Reducing the reliance on nitrogen fertilizer for wheat production. J. Cereal Sci..

[50] Sedlář O., Balík J., Černý J., Peklová L., Kubešová K. (2014). Vliv injektážní aplikace dusíku na příjem zinku a železa ozimou pšenicí a jarním ječmenem. J. Cent. Eur. Agric..

[51] Spiegel H., Sager M., Oberforster M., Mechtler K., Stüger H.P., Baumgarten A. (2009). Nutritionally relevant elements in staple foods: Influence of arable site versus choice of variety. Environ. Geochem. Health.

[52] Lolas G.M., Palamidis N., Markakis P. (1976). The phytic acid-total phosphorus relationship in barley, oats, soybeans and wheat. Cereal Chem..

[53] Liu Z.H., Wang H.Y., Wang X.E., Zhang G.P., Chen P.D., Liu D.J. (2007). Phytase activity, phytate, iron, and zinc contents in wheat pearling fractions and their variation across production locations. J. Cereal Sci..

[54] Etcheverry P., Grusak M.A., Fleige L.E. (2012). Application of in vitro bioaccessibility and bioavailability methods for calcium, carotenoids, folate, iron, magnesium, polyphenols, zinc, and vitamins B 6, B 12, D, and E. Front. Physiol..

[55] Hussain S., Maqsood M.A., Rengel Z., Aziz T. (2013). Biofortification and estimated human bioavailability of zinc in wheat grains as influenced by methods of zinc application. Plant Soil.

